# Observing Maternal Restriction of Food with 3–5-Year-Old Children: Relationships with Temperament and Later Body Mass Index (BMI)

**DOI:** 10.3390/ijerph15061273

**Published:** 2018-06-15

**Authors:** Claire V. Farrow, Emma Haycraft, Jacqueline M. Blissett

**Affiliations:** 1 Department of Psychology, School of Life and Health Sciences, Aston University, Aston Triangle, Birmingham B4 7ET, UK; j.blissett1@aston.ac.uk; 2 School of Sport, Exercise & Health Sciences, Loughborough University, Loughborough LE11 3TU, UK; e.haycraft@lboro.ac.uk

**Keywords:** child feeding, eating, temperament, maternal restriction, controlling feeding, BMI, observation

## Abstract

Overt parental restriction of food has previously been associated with child weight; however, most research has relied on self-reported feeding behaviour, or observations which give little opportunity to observe restriction of food. Using a novel lab-based observational technique to increase the opportunity to observe maternal feeding restriction, we explored the relationships between maternal restriction, child responses to restriction and child temperament with child body mass index (BMI) Z-scores over time. Sixty-two mother child dyads were recruited to the study when their children were aged 3–5 years and were followed up 2 years later (*N* = 39 dyads). Families were observed during a feeding interaction in the laboratory where cookies were offered with the main meal to increase the opportunity for maternal restriction of food. Feeding observations were coded and child temperament and BMI were measured. Controlling for current child BMI Z-scores, greater maternal verbal and physical restriction of food at 3–5 years was related to higher child BMI Z-scores at 5–7 years. More emotional children were less likely to experience restriction and less likely to accept attempts to restrict their food intake. Further research should consider children’s reactions to parental feeding behaviours in greater depth and explore how feeding practices interact with child temperament in the prediction of changes in child weight.

## 1. Introduction

Covert restriction of unhealthy food, or the tendency not to keep high fat and high calorie foods in the home, has been suggested to be protective against unhealthy snacking behaviour in children [1]. However, parental use of overt and overly controlling food restriction is thought to be a counterproductive behaviour which is associated with children eating more in the absence of hunger [2], weight gain [3,4,5], and emotional eating [6]. Overly controlling feeding practices are believed to undermine the child’s ability to recognise their own hunger and satiety signals, thereby eroding the capacity to self-regulate energy needs [7]. In addition, restriction has been shown in experimental paradigms to increase children’s desire for restricted foods [8].

However, findings have been mixed and research with younger children has often failed to replicate the relationships between maternal restriction of food and children’s eating in the absence of hunger [9,10]. Furthermore, research with younger children has reported that maternal reports of restrictive feeding practices are related to *lower* subsequent child body mass index (BMI) Z-scores across infancy [9] and are associated with *less* eating in the absence of hunger in longitudinal research with children at 27 months of age [11]. Discrepancies in the literature may reflect variability in methodologies and participant groups, but children’s responses to parental restriction may be important for determining the impact that restriction has on subsequent eating and weight. For example, it may be that younger children accept parental restriction of food more readily and are less likely to challenge restriction or to seek out desired foods against their parent’s wishes. Older children may be more likely to challenge parental restriction and override attempts to restrict food, both during specific feeding interactions, and also subsequently when left alone with access to food. However, to date, research has failed to explore the impact of a child’s responses to parental restriction of food on later eating behaviour or weight.

Another characteristic which has been poorly explored in relation to the impact of parental restriction of food on child weight is child temperament. A recent systematic review identified evidence for an association between child BMI and aspects of child temperament including poor self-regulation, distress to limitations, soothability, and negative affect [12]. There is a developing evidence base which suggests that aspects of temperament relate directly to behaviours associated with excess food intake [13] and infant emotional distress has been associated with heavier weight across childhood [14]. However, temperament is also likely to influence parental feeding practices. For example, parents may overfeed more distressed children [15], or use more pressuring feeding practices with more unsociable children [16]. A further possibility is that feeding practices may interact with parental perceptions of child temperament in the prediction of child eating and weight. For example, Rollins, et al. [17] have found that the effects of maternal restriction of food differ according to children’s regulatory and appetitive behaviours; with children with lower inhibitory control and higher approach tendencies being more likely to increase food intake in response to maternal restriction.

Most previous research in this area has relied on parental descriptions of their restrictive feeding practices and styles, yet parental reports of feeding practices have been shown to correlate poorly with independent observations of these behaviours at home and in the laboratory [12,18], particularly for mothers of children who are overweight [6]. There is a lack of research using direct observational data to explore how observed feeding restriction impacts on subsequent child eating behaviour or weight. A particular challenge for researchers is to observe parental restriction of food in a specific mealtime interaction, given that it is counterintuitive for parents to offer children food to eat which they then forbid. Furthermore, there has been little observational research examining the different types of restrictive feeding practices that parents use at a mealtime; for example, parents may move food off the plate or away from the child (physical restriction) or they may give instructions or commands about how much of a food can be eaten (verbal restriction). To establish the potential effects of observed restriction on children’s eating and weight gain, a novel mealtime observation paradigm is required which deliberately includes high fat, high sugar foods, which are more likely to be targets for parental restriction [19]. Moreover, whilst observational codes have been developed to assess parental controlling feeding practices during feeding interactions, there exists no observational system to code children’s responses to restriction of food. Children can accept parental verbal or physical restriction of food or can reject parental instruction and continue to consume food. Given the discrepancies identified in previous research exploring the impact of restriction on child weight according to child age, it is important to consider whether child acceptance or rejection of feeding restriction may relate to subsequent child weight.

The first aim of the current study is to explore the efficacy of using a novel approach to observe maternal verbal and physical feeding restriction by providing a commonly restricted food during a laboratory mealtime. The second aim is to evaluate whether observed maternal restriction of food with children aged 3–5 years, and children’s responses to this (i.e., acceptance or rejection of restriction), predict child BMI Z-scores 2 years later. The final aim is to explore whether child temperament at 3–5 years is associated with restriction, children’s responses to restriction, and subsequent child BMI Z-scores. Based on previous literature it is hypothesised that greater maternal verbal and physical restriction of food at 3–5 years, and greater child rejection and lower child acceptance of that restriction, will be correlated with higher child BMI Z-scores at 5–7 years. It was hypothesised that greater rejection and lower acceptance of restriction would correlate with a higher child BMI Z-score. We also hypothesised that children who were more emotional or sociable would be more likely to receive restriction, would be more likely to reject and less likely to accept restriction and would have higher BMI Z-score at 5–7 years. This study uses a longitudinal design, allowing us to explore the prediction of BMI Z-scores over time whilst controlling for baseline child BMI Z-scores.

## 2. Materials and Methods

### 2.1. Participants

A sample of 124 participants (62 mother-child dyads) were recruited to this longitudinal study when the children were aged approximately 3–5 years of age. The retention rate was 63% and 78 participants (39 mother-child dyads) were followed up 2 years later. Participants were recruited via advertisements to parents in the East Midlands area of the UK. Families were eligible to participate if they had a child aged between 3 and 5 years old (time point 1: TP1) with no medical conditions affecting eating or feeding. Families were followed up two years later (time point 2: TP2). The sample comprised 33 boys and 29 girls. The children’s ages ranged from 34–59 months at time point 1 (mean = 46.06 months, SD = 6.81). Most (83%) mothers described their ethnicity as White British and mothers had on average 4 years of post-16 education (SD = 3.01). There was no significant difference between families retained in the study compared to those who dropped out in maternal BMI; t(60) = −0.28, *p* > 0.05, or child BMI Z score; t(60) = −0.05, *p* > 0.05, at time 1. This study was approved by the ethics committee at Loughborough University and registered as a trial at clinicaltrials.gov as NCT01122290. All parents gave informed consent for their child and their self to participate and all children verbally assented to take part. All procedures were conducted in accordance with the Declaration of Helsinki as revised in 1983. The project was reviewed and approved by Loughborough University’s Ethics Approvals (Human Participants) Sub-Committee; Project number: R08-P21.

### 2.2. Procedure

At both time points, families were welcomed to the laboratory and were given an opportunity to familiarise themselves with the room and play with the age-appropriate toys available. After a period of settling in, mother-child dyads were given a standardised lunch at a child friendly table; maternal and child feeding and eating during this time was observed and discretely recorded via video-recording equipment. Because we were interested in recording as typical behaviour as possible, mothers were not instructed to restrict any specific food, but to feed their child as they would normally. Whilst mothers were fully informed that their mealtime interactions were being recorded they were not made explicitly aware that their use of restriction would be a focus of analysis. Following lunch, children were given the opportunity to play with the toys whilst their mothers completed a series of questionnaires. Children and mothers were weighed and measured in light indoor clothing with their shoes removed. Families were paid for their time, thanked and were free to leave.

### 2.3. Measures

*Observations of eating and feeding:* Mothers and children were observed consuming a standardised lunch at time point 1. The child’s lunch consisted of: 1 white bread roll, 1 slice of chicken, 1 slice of cheese, 4 cheese crackers, 3 pieces of chopped apple, 5 carrot sticks and 2 chocolate chip cookies. Restriction is the feeding practice that is observed least during family mealtimes [6,20], therefore we intentionally included the chocolate chip cookies with the child’s meal to increase the opportunity to observe physical and verbal restriction of food. No other forms of non-essential foods were added to the meal as the cookies represented approximately one child sized portion of food and we did not want to present excessive amounts of high-fat, high-calorie foods to ensure that the meal to be as natural as possible for a lunchtime setting. Mothers’ lunches were the same but slightly larger as they included 2 bread rolls, 2 slices of chicken and 2 slices of cheese. Where mothers indicated that they or their child were vegetarian, chicken was replaced with additional cheese. Mothers and children were each provided with a drink of water with their meal. Mothers and children were asked to eat from their own plates until they felt full and could ask for additional food if they wished (no families requested more food).

Observations of maternal restriction with food were coded using the Family Mealtime Coding System (FMCS) [18] which assesses various feeding practices including Verbal Restriction of Food (e.g., “Don’t eat that cookie now”) and Physical Restriction of Food (e.g., removal of a food from the child’s plate). The frequency that each of these feeding practices was observed was recorded and a total score for each subscale was created for each parent. In addition, based on previous research which has highlighted the importance of observing parent and child responsiveness to each other [21], we coded for children’s responses to parental restriction. Specifically, we coded whether children accepted or rejected any parental attempts to restrict food intake. Acceptance of restriction was defined as not eating restricted food, allowing the caregiver to remove restricted food and following instructions to stop eating. Rejection of restriction was defined as ignoring requests to not eat restricted food or making attempts to eat food which was restricted or removed from the plate (verbally or physically). Codes were computed as raw scores of the number of instances of acceptance and rejection. In order to factor in the potential effects of the frequency of maternal restriction behaviour on the child’s opportunity to react to restriction, percentage scores were also calculated to indicate acceptance/rejection of restriction as a proportion of the use of maternal restriction (count/maternal use of restriction * 100). Feeding observations were coded by a research associate trained to use the FMCS, 25% of observations were coded by a second trained coder and inter-rater reliability was calculated as the total agreement on scores between raters. Consistency was good, with 60–80% exact agreement on the scores provided for the restriction subscales.

*Child temperament:* The Emotionality Activity Sociability (EAS) temperament questionnaire [22] was used to measure child emotionality and sociability. These subscales were used based on previously identified relationships between child emotionality [12,23] and sociability [24] with eating behaviour. Emotionality can be defined as whether the child becomes aroused easily and intensely and sociability reflects how much the child prefers to be with others compared to alone. The questionnaire is coded using a 5-point Likert scale with higher mean scores indicating a great presence of each temperament trait. The EAS is suitable for children aged 1–9 years, is a well-used measure with good stability and factor structure and relates well to other measures of child temperament [25].

*Body Mass Index (BMI) scores.* Children were weighed and measured in light indoor clothes without shoes at both T1 and T2. Weight and height scores were converted to BMI Z-scores using the Child Growth Foundation Reference Curves which adjust for the child’s gender and age. This package is based on the LMS data computations [26,27]. Mothers were also weighed and measures in light indoor clothes with shoes removed at both time points. BMI was calculated as kg/m^2^.

### 2.4. Data Analysis

Pearson’s correlations were used to explore whether child age was correlated with maternal feeding restriction or child acceptance or rejection of such restriction. There were no significant correlations between child age (in months) and maternal use of restrictive feeding practices (verbal or physical restriction) or children’s acceptance or rejection of restrictive feeding practices. Child age in months was positively correlated with emotionality (r = 0.37, *p* < 0.05) but not child sociability (r = −0.15, *p* > 0.05). Independent sample *t*-tests were used to explore whether there were differences between boys and girls in their BMI Z-scores. There were no significant differences between boys and girls in terms of their BMI Z-score at time point 1 (TP1); t(60) = 0.84, *p* > 0.05, or time point 2 (TP2); t(38) = 1.99, *p* > 0.05, and there was no significant correlation between the number of siblings a child had and their BMI Z score at TP1 (r = 0.06, *p* > 0.05) or TP2 (r = 0.04, *p* > 0.05). Pearson’s correlations were also used to explore whether maternal education and maternal BMI were related to child BMI Z-scores at TP1 or TP2. Maternal years of education post-16 was not significantly correlated with child BMI Z-score at TP1 (r = −0.13, *p* > 0.05) or TP2 (r = 0.24, *p* > 0.05). Maternal BMI at TP1 was not significantly correlated with child BMI Z-scores at TP1 (r = 0.22, *p* > 0.05), but maternal BMI at TP2 was significantly correlated with child BMI Z-scores at TP2 (r = 0.35, *p* < 0.05). In light of this, and other research suggesting that parental weight status increases children’s susceptibility to weight gain as a result of feeding restriction [5], maternal BMI at TP2 was controlled for in further analyses with child BMI.

A series of partial correlations were performed to explore whether observed maternal feeding restriction, child acceptance or rejection of that restriction as a percentage of maternal restriction, and child temperament at TP1 were correlated with subsequent measures of child BMI Z-scores at TP2, after controlling for child BMI Z-scores at TP1 and maternal BMI at TP2. Correlations were also used to explore whether child emotionality and sociability were correlated with observed maternal feeding restriction, child acceptance or rejection of that restriction as a percentage of maternal restriction, and child BMI Z-scores. Pearson’s correlations were used to analyse relationships with sociability, whilst partial correlations were used (controlling for child age) to analyse correlations with emotionality. All correlations were 1-tailed due to the directional nature of the hypotheses and significance was considered at *p* ≤ 0.05.

## 3. Results

Mean maternal BMI was 24.76 at TP1 (SD = 5.01) and 25.40 at TP2 (SD = 5.13) suggesting the sample were, on average, borderline overweight and overweight at time point 2. Mean child BMI Z-score was 0.09 at TP1 (SD = 1.00) and −0.04 at TP2 (SD = 0.92) indicating that children had a healthy BMI score on average. The mean duration of mealtime observations was 21.33 min (SD = 6.54) and children consumed an average of 48.17 mouthfuls (SD = 19.25). The mean ratings for maternal observed verbal and physical feeding restriction are reported in Table 1. The sample size for the mean scores is 39 participants, except for the percentage scores which only included dyads where there was evidence of restriction of food (*N* = 16 verbal restriction and *N* = 11 physical restriction). The means are similar to other observations of feeding behaviour with children in this age range [18,28]. The mean acceptance of verbal and physical restriction was greater than the mean rejection of restriction suggesting that children conceded to mothers’ attempts to restrict their food intake more often than they over-rode such attempts. When acceptance and rejection were computed as a percentage of the mother’s behaviour, children accepted maternal restriction 67–70% of the time (therefore rejecting maternal restriction 30–33% of the time).

**Figure 1:**
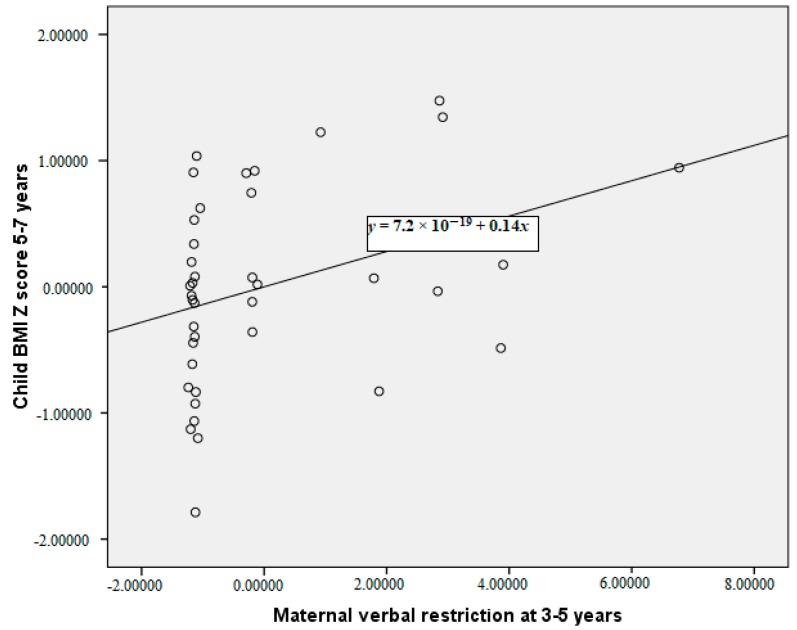
Partial correlation between maternal verbal restriction at 3–5 years and child body mass index (BMI) Z-score at 5–7 years, controlling for child BMI Z-score at 3–5 years (plot of the unstandardised residuals).

**Figure 2:**
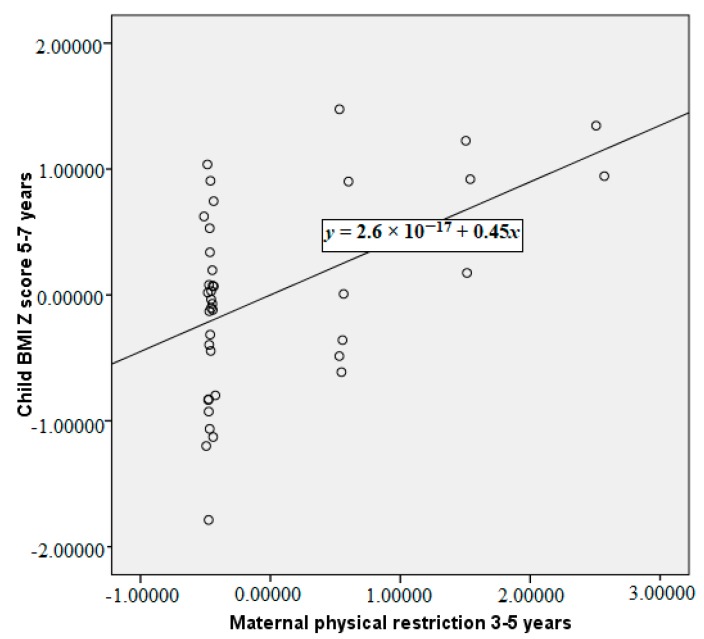
Partial correlation between maternal physical restriction at 3–5 years and child BMI Z-score at 5–7 years, controlling for child BMI Z-score at 3–5 years (plot of the unstandardised residuals).

**Figure 3:**
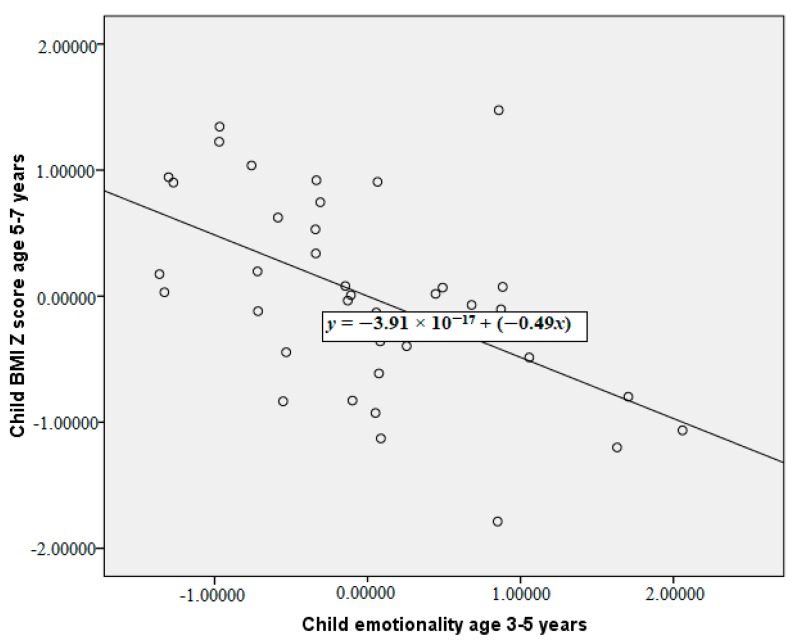
Partial correlation between child emotionality at 3–5 years and child BMI Z-score at 5–7 years, controlling for child BMI Z-score at 3–5 years (plot of the unstandardised residuals).

The partial correlations between maternal restriction of food, child acceptance or rejection of that restriction, and child temperament at 3–5 years with subsequent child BMI Z-scores 2 years later are reported in Table 2. These correlations are reported having controlled for baseline child BMI Z-scores and for maternal BMI. Greater maternal verbal and physical restriction of food at 3–5 years were both significantly correlated with a higher subsequent child BMI Z-score at 5–7 years. Our primary analysis of the child acceptance/rejection of restriction data indicates that when considered as a proportion of maternal restriction there was no significant relationship between acceptance/rejection of restriction and child BMI Z-score. More emotional child temperament at 3–5 years was associated with a lower child BMI Z-score at 5–7 years. More emotional children were significantly less likely to experience physical restriction of food, they were also less likely to accept maternal physical restriction. The significant relationships between maternal verbal restriction, physical and child emotionality at 3–5 years with child BMI Z-score at 5–7 years are plotted in Figure 1, Figure 2 and Figure 3, controlling for child BMI z-score at 3–5 years using unstandardised residuals. There were no other significant correlations between child temperament and observed feeding behaviour.

## 4. Discussion

This study uses a novel observational method in the laboratory to heighten the opportunity for maternal restriction of food to occur and tracks the relationship between restriction, temperament and subsequent child BMI. By controlling for baseline child BMI Z-scores, the findings indicate that mothers who use more verbal and physical restriction in a feeding interaction away from the home when their children are aged 3–5 years are more likely to see greater weight gain in their children across the following 2 years. Moreover, by coding for children’s responses to feeding restriction we were able to establish that children’s acceptance/rejection of restriction as a percentage of the amount of restriction that the child was exposed to was not related to subsequent BMI Z-score. This study also evaluated whether maternal feeding restriction and child temperament were related. Greater child emotionality was correlated with lower subsequent child BMI Z-scores; more emotional children were significantly less likely to be physically restricted from eating food, and if they did experience such restriction, they were significantly more likely to reject attempts to restrict their food intake.

Although previous research has repeatedly demonstrated that overt restriction of food is associated with weight gain across childhood [3,5], most research in this field has relied on self-reported parental feeding practices or observations of natural mealtime interactions where it is rare for parents to offer foods which they then restrict [20]. The findings of this study suggest that, irrespective of the child’s current weight status, greater observed maternal use of verbal and physical restriction of food in the laboratory predict greater child weight gain over the following 2 years. These findings should be considered amongst a growing body of literature which suggests that overt restriction of food may hinder children’s ability to regulate their energy needs over time [8]. By providing a commonly restricted high fat, high sugar food as part of the child’s observed mealtime, we have been able to code both the quantity and type (physical/verbal) of maternal use of restriction of food, in addition to the child’s responses to this restriction. This observational method appears to have been effective at eliciting feeding restriction, with mothers verbally restricting food 1.15 times on average during the observation (with a range from 0–8 instances of verbal restriction), and physically restricting food on average 0.46 times during the observation (with a range from 0–3 instances of physical restriction). These findings suggest that mothers use verbal restriction more often than physical restriction with their children at 3–5 years, but that both verbal and physical restriction are correlated with a subsequently higher child BMI Z-score when children are 5–7-years-old.

Our novel observational paradigm also allowed us to code for children’s responses to any feeding restriction and we found that around one third of the time children refused maternal attempts to restrict their food intake; specifically, on average children rejected verbal restriction of food 33% of the time and rejected physical restriction of food 30% of the time. Importantly the tendency to accept or reject restriction was unrelated to the child’s age suggesting that 5-year-old children are no more resistant to controlling restrictive feeding practices compared to 3-year-old children. However, this is a narrow age range and using a sample of children across childhood may provide different findings as children may become more likely to challenge (or accept) parental feeding control. Children’s reactions to maternal feeding restriction also significantly predicted their subsequent weight trajectory. By controlling for baseline BMI Z-scores at 3–5 years (time 1) in the prediction of weight at 5–7 years (time 2), we are able to establish whether acceptance of restriction is related to changes in child weight. The findings suggest that child acceptance and rejection of physical restriction, and child rejection of verbal restriction of food at time 1 as raw scores were all associated with greater weight gain to time 2. However, when these behaviours were considered as a percentage of the amount of restriction that the children were exposed to, relationships between acceptance/rejection of restriction and later BMI Z-scores were no longer significant. This suggests that the frequency of maternal restriction, and the resulting opportunity to accept or reject restriction, is driving the relationship between children’s responses and their BMI Z-scores.

The findings of this study replicate previous research which has shown that temperament relates to child eating behaviour, BMI and feeding practices [12,13,15,16,23]. Specifically, we found that child emotionality at 3–5 years was correlated with subsequently lower child BMI Z-scores at age 5–7. Infants who are rated to be more ‘difficult’ are also reported to have more negative mealtimes [29]. Haycraft et al. [23] demonstrated that more emotional children exhibit less enjoyment of food and show more food avoidance, which may help to explain longer-term lower BMI Z scores in more emotional children in our sample. Parents also report higher levels of negative affect in picky versus non-picky eaters [30,31], which is in turn associated with lower BMI. We did not examine the effects of food avoidance/pickiness on the relationships in this study, so further work is needed to disentangle possible interactive effects of temperament, child eating behaviour and feeding practice on weight gain. Nonetheless, these data also demonstrate the potential effect of temperament on feeding practices which may moderate weight gain over time. What is novel about this study is the finding that more emotional children were physically restricted with food less often by their caregivers, and that if they were restricted, they were less likely to accept such restriction. Thus, parents of more emotional children may be less likely to perceive the need to restrict food intake because of lower BMI or more picky eating, or may be disinclined to use restrictive feeding practices because of defiance/lack of compliance and/or negative emotional reactions from their children to such practices. Thus temperament, both through eliciting specific feeding practices and affecting children’s responses to parental feeding practices, may result in shaping of both parent and child behaviours that have longer term impact on child BMI Z scores.

In this study we utilised a new approach to observe maternal feeding restriction and this appears to have been effective at capturing instances of verbal and physical restriction of food, as well as children’s responses to such mealtime behaviours. This observational method is novel and provides rich data about the interactive nature of feeding interactions. The longitudinal design of this study is a clear strength which allows us to control for baseline child BMI Z-scores when exploring relationships over time. However, the sample size utilised in this study is small, particularly when children’s responses were considered as a percentage of maternal restriction. The sample is self-selecting and involves predominantly White, middle-class participants and the findings should therefore be treated with caution. We are also not powered to explore how physical and verbal restriction of food may interact to predict child weight or to consider whether maternal restraint and disinhibition may have influenced feeding behaviour and social correctness (e.g., pressure to restrict the non-healthy food item). Further research is required with other, broader and larger groups in order to be able to explore these relationships and generalise these findings, particularly given that research suggests that the relationship between feeding and eating may vary according to culture [32]. Future research should also consider using a greater number of observations of mealtime interactions across childhood, and taking average scores from repeated observations, to allow for a more in depth understanding of how feeding restriction (and children’s responses to such restriction) evolve over time and relate to child eating behaviour and weight gain in the longer term. It would be potentially valuable to code in more detail which foods are restricted verbally and physically in buffet settings by caregivers and explore how children’s responses differ according to the restriction of different food types. Further research should also consider the role of feeding interactions and the potential impact of restriction in other settings away from the primary caregiver (e.g., at nursery, at school or with other caregivers).

## 5. Conclusions

This study provides a novel method for observing maternal feeding restriction and an effective method for coding this behaviour in depth, as well as children’s responses to this feeding behaviour. This study indicates that maternal restriction of food at 3–5 years is associated with heavier child weight at 5–7. The findings further demonstrate that more emotional children are less likely to experience physical restriction of food, are less likely to accept physical restriction of their food and show lower subsequent BMI Z-scores. Further research is needed to understand the different *mechanisms* by which parental restriction and child responses to that restriction interact to predict the development of child eating behaviour and BMI over time.

## Figures and Tables

**Table 1:** Descriptive statistics for observed restriction and child temperament at 3–5 years (T1).

Observed Restriction Behaviours	*N* = 39 ^b^
	Mean (Min–Max)
Maternal verbal restriction of food	1.15 (0–8)
Child acceptance of verbal restriction (count)	0.74 (0–5)
Child rejection of verbal restriction (count)	0.41 (0–6)
Child acceptance of verbal restriction (percentage)	66.56 ^a^ (1–100)
Maternal physical restriction of food	0.46 (0–3)
Child acceptance of physical restriction (count)	0.31 (0–2)
Child rejection of physical restriction (count)	0.15 (0–2)
Child acceptance of physical restriction (percentage)	69.70 ^a^ (0–100)
**Child temperament:**	
Emotionality	2.33 (1–4.40)
Sociability	3.56 (2.6–5)

^a^ scores reflect percentages of the total number of instances of maternal restriction. ^b^ *N* = 39 except for % scores which were only computed when there was evidence of any maternal restriction of food (*N* = 16 verbal restriction and *N* = 11 physical restriction).

**Table 2:** Correlations between observations of restrictive feeding and child temperament at 3–5 years with child BMI Z-scores at 5–7 years.

Child Weight and Temperament	Child BMI Z-Score (5–7 Years) ^a^ *N* = 39 ^d^	Child Emotionality ^b^ (3–5 Years) *N* = 39 ^d^	Child Sociability ^c^ (3–5 Years) *N* = 39 ^d^
**Observed restriction behaviours (child age 3–5 years):**			
Verbal restriction by mother	0.34 *	−0.18	−0.05
Child acceptance verbal restriction (count)	0.26	−0.09	−0.03
Child rejection verbal restriction (count)	0.270 *	−0.19	−0.04
Child acceptance verbal restriction (%)	−0.14	−0.14	−0.04
Physical restriction by mother	0.47 **	−0.35 *	−0.10
Child acceptance physical restriction (count)	0.43 **	−0.29 *	−0.04
Child rejection physical restriction (count)	0.29 *	−0.23	−0.14
Child acceptance physical restriction (%)	0.13	−0.05	0.35
**Child temperament at child age 3–5 years:**			
Emotionality	−0.53 **	-	-
Sociability	−0.16	-	-

1-tailed * *p* ≤ 0.05, ** *p* < 0.01; ^a^ Partial correlations controlling for child BMI Z-score at age 3–5 and for maternal BMI. ^b^ Partial correlations controlling for child age in months. ^c^ Pearson’s correlations. ^d^ *N* = 39 except for % scores which were only computed when there was evidence of any maternal restriction of food (*N* = 16 verbal restriction and *N* = 11 physical restriction).
